# Macrophages lift off surface-bound bacteria using a filopodium-lamellipodium hook-and-shovel mechanism

**DOI:** 10.1038/srep02884

**Published:** 2013-10-07

**Authors:** Jens Möller, Tessa Lühmann, Mamta Chabria, Heike Hall, Viola Vogel

**Affiliations:** 1 Laboratory of Applied Mechanobiology, Department of Health Sciences and Technology, ETH Zurich, CH-8093 Zurich, Switzerland

## Abstract

To clear pathogens from host tissues or biomaterial surfaces, phagocytes have to break the adhesive bacteria-substrate interactions. Here we analysed the mechanobiological process that enables macrophages to lift-off and phagocytose surface-bound *Escherichia coli* (*E. coli*). In this opsonin-independent process, macrophage filopodia hold on to the *E. coli* fimbriae long enough to induce a local protrusion of a lamellipodium. Specific contacts between the macrophage and *E. coli* are formed via the glycoprotein CD48 on filopodia and the adhesin FimH on type 1 fimbriae (hook). We show that bacterial detachment from surfaces occurrs after a lamellipodium has protruded underneath the bacterium (shovel), thereby breaking the multiple bacterium-surface interactions. After lift-off, the bacterium is engulfed by a phagocytic cup. Force activated catch bonds enable the long-term survival of the filopodium-fimbrium interactions while soluble mannose inhibitors and CD48 antibodies suppress the contact formation and thereby inhibit subsequent *E. coli* phagocytosis.

Bacterial phagocytosis by immune cells is a crucial step in the host defence against microbial invaders. During clearance of the pathogens from the host tissue, immune cells often encounter sessile bacteria bound to biomedical implants, to extracellular matrix or to cell surfaces. Macrophages, as major players of the host innate immune system, play an important role during the host response to acute and chronic inflammations^1^ as it might occur during wound healing^2^, biomaterial-related or urinary tract infections^3^,^4^. Additionally, they perform important tissue surveillance functions and mature tissue residing macrophages police their immediate surroundings to identify and clear pathogens, cell debris and foreign particles from the host^5^. While many of the molecular players involved during phagocytosis have been well characterized^6^, the mechanical aspects how macrophages can create sufficient forces to lift bacteria off surfaces have not yet been described, neither *in vivo* nor *in vitro*.

Parts of the sequential events of pathogen phagocytosis are well characterized and share a common sequence: once recruited to the site of infection, macrophages are activated and identify potential harmful pathogens by specific receptors^7^. Upon receptor-mediated binding of the target, the local actin cytoskeleton is remodelled and the phagocytic cup is formed^8^. Through actin-mediated protrusions of the macrophage membrane, the target can be internalized^9^. Since little is known regarding the mechanical aspects involved, the focus of this study was to analyse the biomechanics by which macrophages lift-off tightly bound *Escherichia coli* (*E. coli*) from surfaces.

While most strains of *E. coli* are harmless, enterohemorrhagic *E. coli* (EHEC) and uropathogenic *E. coli* (UPEC) can cause life-threatening infections upon entry into the blood circulation through lesions of the digestive track or the epithelium of the urinary tract^10^ respectively. Analysing this process is physiologically relevant as macrophages frequently encounter bacteria that are tightly bound to (engineered) surfaces^11^ or to ECM fibrils^12^. Besides the physicochemical properties of the material and the bacterial surfaces^13^, external mechanical force can regulate the strength of bacterial adhesive bonds. While most receptor-ligand interactions are known to dissociate faster under tensile forces (slip bonds), it is well established that the *E. coli* adhesin FimH forms long-lived catch bonds with mannoses, i.e. bonds that are activated by mechanical force (for reviews see^14^,^15^).

In this single-cell analysis study, we describe kinetic and mechanistic details of a multistep process that enables macrophages to pick up surface-adhering type 1 fimbriated *E. coli* in an opsonin-independent, but mannose-specific manner. To specifically recognize type 1 fimbriated *E. coli*, macrophages use the mannose-presenting glycosylphosphatidylinositol (GPI)-anchored plasma membrane receptor CD48^16^. Since each *E. coli* type 1 fimbrial tip presents just a single FimH adhesin^17^, and thus a single mannose-binding pocket, each fimbrium can engage with just one single CD48 receptor. We show here that filopodia retraction is not sufficient to lift-off surface bound *E. coli* and that the mechanical interplay of forming a long-term bond with a filopodium and subsequent lamellipodium protrusion is required for the pickup that initiates phagocytosis.

## Results

To allow for co-adhesion of *E. coli* (UPEC strain J96) and macrophages (J774.1), we performed all phagocytosis experiments on glass substrates coated with a mixture of purified human plasma fibronectin (FN) and the glycoprotein Ribonuclease B (RNaseB). The extracellular matrix protein FN promoted integrin-mediated macrophage adhesion while the tri-mannose motifs on RNaseB facilitated FimH-mediated adhesion of type-1 fimbriated *E. coli*^18^. Polysaccharides are present on the glycocalyx of many endothelial and epithelial cells and the glycopattern of RNaseB^19^ is similar to uroplakin 1a, the physiological receptor of UPECs on the uroepithelium^20^.

### Macrophages use filopodia to sense and form long-lived interactions with surface-adhering bacteria

**Figure 1:**
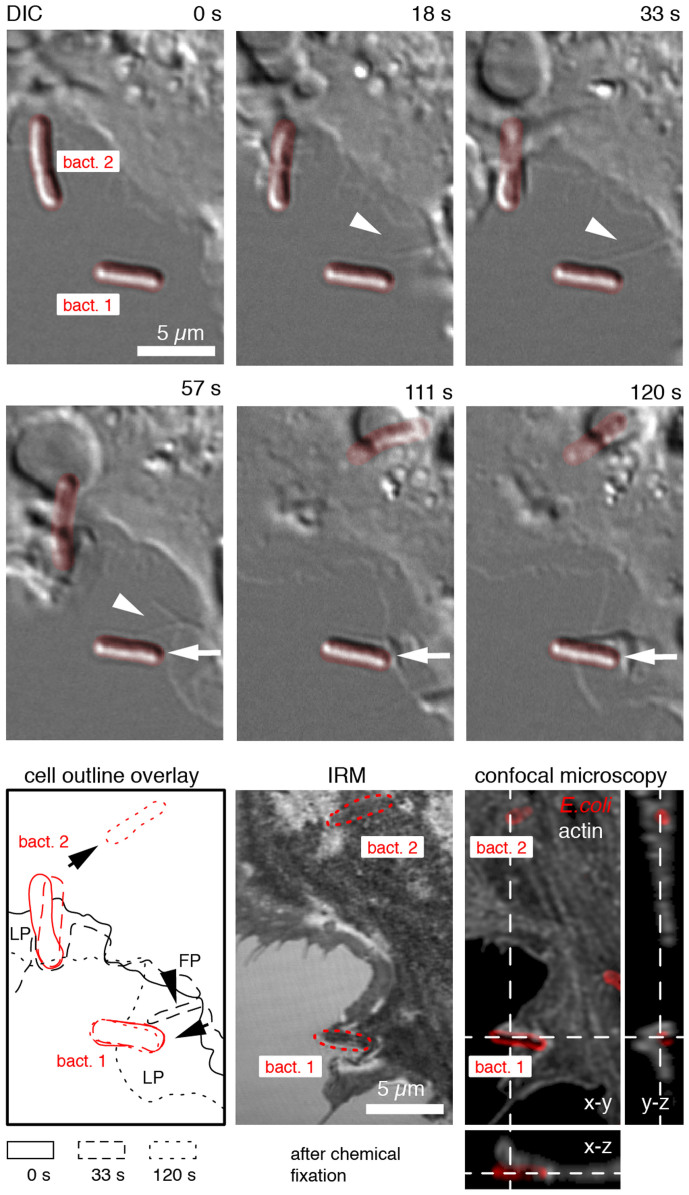
Multistep macrophage uptake of surface-bound *E. coli*. Macrophage encountered surface-bound *E. coli* (bact.1, bact.2, false coloured red) by filopodia (FP, arrowhead)) and lamellipodia (LP, arrow) as visualized by live cell DIC microscopy (Supplementary Movie 1). For bacterium 1, the initial FP contact remained intact sufficiently long for the macrophage to locally protrude a LP towards the bacterium (arrow, 33–57 s). Upon LP contact, the membrane locally ruffled in front of the bacterium (111 s) followed by LP protrusion under the bacterium (120 s). LP protrusion underneath the bacterium was confirmed by interference reflection microscopy (IRM) and 3D confocal fluorescence microscopy of the same cell after chemical fixation at 120 s. Bacteria were labelled with primary anti-*E. coli* and secondary Dylight 649 antibody (red). The macrophage F-actin cytoskeleton was stained with Alexa-488 phalloidin (white). Confocal stacks were deconvolved using Huygens software. For bacterium 2, the LP was already in contact at the start of the time series. The uptake of bact. 2, as indicated by the rapid displacement of the bacterium (57–120 s, see outline overlay) was confirmed by 3D confocal microscopy (y-z cross section, 120 s). Due to the fast macrophage dynamics, the last frame of the DIC sequence (120 s) does not overlay exactly the IRM as well as with the confocal micrograph of the fixed sample.

To analyse the dynamics of macrophage-bacteria uptake at high spatial and temporal resolution, we combined live cell DIC time-lapse microscopy (Δt = 3 s) with interference reflection (IRM) and 3D confocal fluorescence microscopy and observed a multistep macrophage uptake of sessile *E. coli*. In the depicted representative sequence of events (Fig. 1), the macrophage extended filopodia to explore the microenvironment and to bind to surface adhering type 1 fimbriated *E. coli* (Fig. 1, bact. 1, 0–33 s, Supplementary Movies 1 and 2). With a filopodium contact formed (33 s), the macrophage locally protruded a lamellipodium towards the bacterium (33–57 s). Upon contact, the lamellipodium deformed (57–111 s) before it protruded underneath the bacterium (111–120 s). To confirm that the lamellipodium went underneath the bacterium, the sample was chemically fixed after 120 seconds with 4% paraformaldehyde. IRM and confocal fluorescence microscopy of the fixed sample showed that the macrophage membrane engulfed rather than spread over the bacterium (Fig. 1, bact.1, IRM, confocal microscopy, x-z and y-z cross sections). From the start of the DIC time series, a second bacterium (Fig. 1, bact. 2) was in contact with the macrophage lamellipodium. The macrophage membrane rapidly engulfed the bacterium (0–33 s) followed by a displacement from its initial spot on the glass substrate (Fig. 1, cell outline overlay; 57–120 s). The y-z cross section of the reconstructed confocal stack confirmed that the bacterium (bact. 2) was internalized by the macrophage.

**Figure 2:**
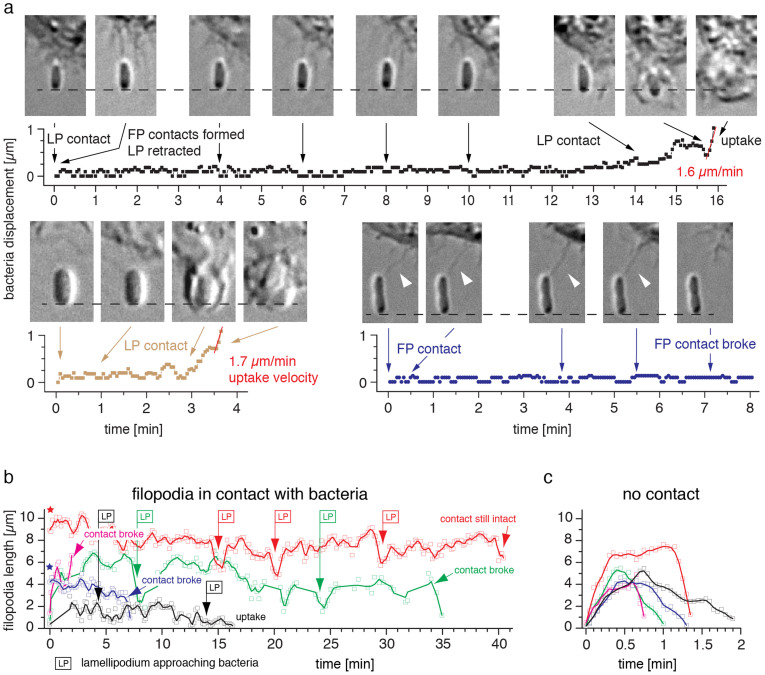
Formation of long-lived interactions between filopodia and surface-bound *E. coli*. (a) Kinetic analysis of bacterial displacements during filopodia (FP) contact and lamellipodia (LP) uptake. Upper row: With intact filopodia-bacterium contacts, no substantial bacterial displacement was observed for 14 min (Supplementary Movie 4, see Methods for displacement analysis). Only after the lamellipodium contacted the bacterium, it was displaced from its original position and the uptake proceeded with 1.6 μm/min. Lower row left: Bacterial displacement initiated by a direct lamellipodium contact without detection of a previous filopodium contact (Supplementary Movie 5). Uptake proceeded with 1.7 μm/min. Lower row right: A filopodium contact alone was not sufficient to displace and pick up a bacterium that firmly adhered to the surface (Supplementary Movie 6). (b) Kinetic analysis of individual filopodium-bacterium contact lengths. 5 representative traces are plotted, including the filopodium-bacterium contacts analysed in (a) (black and blue traces, Supplementary Movies 2 and 3). Long-lived interactions of up to 40 min were observed. The onset of a lamellipodium protrusion towards the captured bacterium is indicated (LP). Note that some contacts were already present at the start of the time series (asterisk). Lines represent adjacent 3-point averages. (c) Extension and withdrawal traces of individual filopodia that were not in contact with surface-bound *E. coli* (Supplementary Movie 2). If no contact was formed, filopodia retracted within 2 minutes.

To further analyse the interplay of the filopodia-*E. coli* contacts and the lamellipodium uptake, we analysed the displacement of the bacteria after initial macrophage encounter from 15 independent DIC live cell time-lapse experiments. We observed in all movies that a filopodium contact alone was not able to displace bacteria (Fig. 2A). Only upon protrusion of a lamellipodium, the bacteria were picked up from the surface (Fig. 2A, Supplementary Movies S2 and S3). Although the filopodia contacts were not sufficient to lift-off the bacteria, they remained intact for up to 40 minutes (Fig. 2B, Supplementary Movie S3). Out of 25 randomly chosen filopodia-*E. coli* contacts, 15 contacts remained intact for at least 16 minutes while the filopodia typically retracted in less than 2 minutes (Fig. 2C). Three newly formed contacts broke within 4 minutes. All other contacts were either present at the start or still intact at the end of the time-lapse acquisition. We typically observed 5 subgroups of filopodia-*E. coli* contacts (Fig. 2B): (i) stable contacts formed with subsequent lamellipodia uptake (black, Supplementary Movie S4), (ii) stable contacts formed that broke before lamellipodia uptake (green, Supplementary Movie S6), (iii) transient contacts formed that broke within 4 minutes (purple), (iv) contacts already present at start (blue) and (v) contact stable over entire acquisition time. The macrophage-*E. coli* contacts commonly formed by filopodia extension. However, we also observed highly motile lamellipodia that encountered the surface-bound bacteria without previous filopodium contact (Fig. 2A, Supplementary Movie S4). Upon retraction of these lamellipodia, filopodia-like macrophage-bacteria contacts were formed. These contacts showed similar lifetimes when compared to the contacts made by filopodia directly.

### Mannosylated CD48 on the macrophage plasma membrane can form long-lived molecular contacts with the bacterial adhesin FimH located at the fimbrial tip

**Figure 3:**
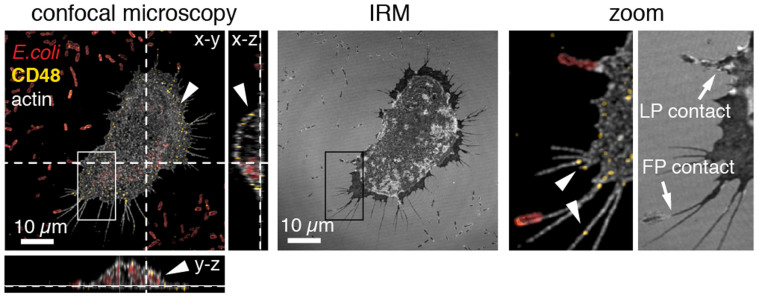
Immunostaining of the GPI-anchored mannosylated FimH receptor CD48 on the J774A.1 macrophage cell membrane. Deconvolved confocal fluorescence and interference reflection micrographs (IRM). Samples were stained for macrophage actin (white), CD48 (yellow) and *E. coli* (red). CD48 exclusively localized within the macrophage membrane including filopodia (see arrowheads and x-z and y-z cross sections). Filopodia (FP) and lamellipodia (LP) – bacteria contacts were suggested by IRM.

To identify the receptor-ligand interaction that is responsible for the long-lasting filopodia-*E. coli* interaction, we stained for the GPI-anchored receptor CD48. CD48 has previously been reported as FimH-specific mannose-sensitive *E. coli* receptor in human brain microvascular endothelial cells and mast cells^21^,^22^. Immunostaining with monoclonal CD48 antibody prior to chemical fixation in 4% paraformaldehyde confirmed that CD48 localizes on the plasma membrane, including filopodia, of the murine J774A.1 macrophages used in this study (Fig. 3, arrowhead). Interference reflection microscopy (IRM) and confocal fluorescence imaging suggest the existence of direct filopodia (FP) and lamellipodia (LP) contacts with surface-bound *E. coli* (Fig. 3, arrow).

**Figure 4:**
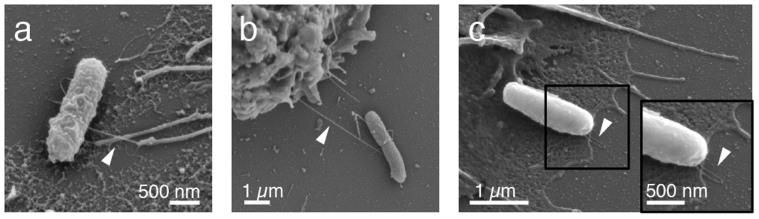
Macrophage - *E. coli* contacts as seen in SEM micrographs. (a) Macrophage filopodium bound to an individual *E. coli* type 1 fimbrium. (b) Long-distance macrophage – bacterium contact (c) Lamellipodium localized underneath a surface-bound bacterium. The arrowhead points towards the last two fimbriae that are still in contact with the mannosylated surface.

**Figure 5:**
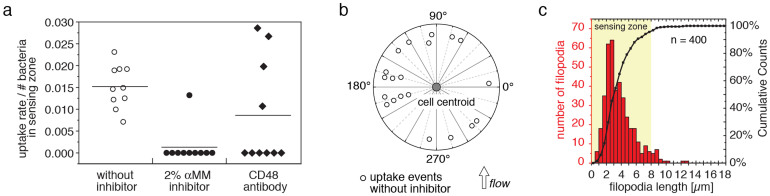
Bacterial uptake was blocked by mannose inhibitor, was substantially reduced by CD48 antibodies and was independent of the direction of fluid flow. (a) Addition of 2% soluble α−D-mannopyranoside inhibitor (αMM) and CD48 antibodies substantially reduced the rate of *E. coli* phagocytosis. Uptake rates were analysed for 10 independent macrophages during 10 min live cell experiments and normalized by the time-averaged number of bacteria available in the zone explored by filopodia. Mean values are given as horizontal line. (b) Bacterial uptake was observed at all sides of the macrophages and was independent of the direction of fluid flow (0.1 ml/min flow rate/0.06 pN/μm^2^ shear stress). (c) Filopodia length distribution as analysed from fixed macrophages. 95% of the filopodia (n = 400) have a length less than 8 μm, which we defined here as the filopodia sensing zone (yellow area).

Due to the limited optical resolution, the existence of direct specific contacts of single macrophage filopodia with *E. coli* type 1 fimbriae was verified by scanning electron microscopy (Fig. 4A). To confirm that the filopodia-fimbriae bonds were mediated by specific mannose-sensitive FimH-CD48 interactions, 2% soluble methyl α-D-mannopyranoside (αMM) was added to the medium prior to macrophage injection for live cell DIC microscopy. Upon the addition of the mannose inhibitor, phagocytosis of type 1 fimbriated *E. coli* within the reach of filopodia was blocked without changing the macrophage filopodia dynamics (Fig. 5A, C). The soluble inhibitor thus competed with CD48 for the FimH mannose-binding pocket. Likewise, when the macrophages were incubated with CD48 antibodies prior to the experiment, the bacterial uptake rate was reduced (Fig. 5A). Yet, the reduction in bacterial phagocytosis was not as pronounced as observed for the soluble mannose inhibitor, which was expected since the antibody epitope on CD48 is not known and therefore the mannose residues on CD48 might still be accessible for FimH. Additionally, bacterial uptake was not impacted by media flow and occurred in all directions around the macrophage cell circumference (Fig. 5B).

**Figure 6:**
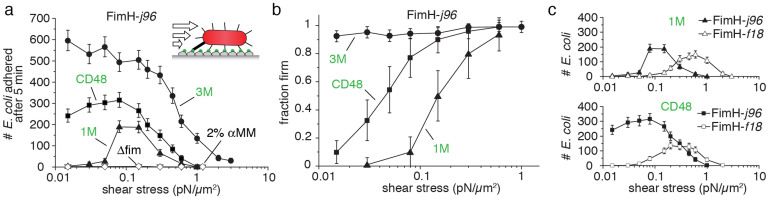
Shear stress dependent FimH-specific *E. coli* adhesion to mannosylated CD48 surfaces. (a) Accumulation of type I fimbriated *E. coli* FimH-j96 bacteria on flow chamber bottom glass surfaces coated with CD48, mono-mannose bovine serum albumin (1 M) and tri-mannose RNaseB (3 M) under varying shear stresses τ (1 pN μm^−2^ = 1 Pascal or 10 dynes cm^−2^). *E. coli* accumulation was analysed after 5 min. As negative controls, either 2% of a mono-mannose inhibitor (αMM) was added to the media (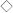), or the non-fimbriated *E. coli* parent strain AAEC191A was used (Δfim, 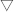). (b) Fraction of *E. coli* FimH-j96 that adhered firmly on CD48, 1 M and 3 M. Bacteria were defined firmly adhering if they moved less than one-half of a bacterial diameter over >30 s. (c) Effect of different FimH variants on bacterial accumulation to 1 M and CD48. While the low binding FimH-f18 strain only adhered to CD48 and 1 M above a critical shear stress, FimH-j96 *E. coli* accumulated on CD48 without any shear threshold.

Since little is known regarding the nature of the oligomannose motif of CD48 and how mechanical force might modulate the CD48-FimH bonds, we studied the shear stress dependent adhesion of *E. coli* to CD48 coated surfaces (Fig. 6). We compared the results to previous FimH catch bond studies on mono- and tri-mannose surfaces (14,18,23,24, Fig. 6A, Supplementary Table S1) and analysed the fraction of rolling versus firmly adhering bacteria to CD48 coated surfaces (Fig. 6B). Adhesion of uropathogenic FimH-j96 *E. coli* to CD48 mannose motifs was more similar to the catch bond adhesion previously observed on tri- rather than mono-mannose^18^ indicating an oligomannose motif on CD48. Adhesion to CD48 was inhibited by the addition of mono-mannose inhibitor (2% αMM). Likewise, the non-fimbriated *E. coli* control strain AAEC191A was unable to adhere to CD48 coated surfaces. Similar to uropathogenic FimH-j96 *E. coli*, accumulation of intestinal FimH-f18 *E. coli* to CD48 was enhanced by shear stress but occurred at higher shear levels (Fig. 6C), which was similar to what has been reported for *E. coli* adhesion to mono-mannose^25^.

### Directed by long-lived filopodia-fimbriae contacts, macrophages locally protrude lamellipodia to lift off bacteria from mannosylated surfaces

In fibroblasts and macrophages, filopodia have been reported to act as tentacles to pull potential targets towards the cell body^26^,^27^. However, in our live cell DIC movies (Supplementary Movies 1, 2 and 3), no inward movement of the sessile surface-adhering *E. coli* mediated by filopodia contacts was observed (Fig. 2A). Since bacteria are attached to the surface via many bonds in parallel that would have to be broken simultaneously^28^, the forces exerted via single filopodia-fimbriae contacts were not sufficient to lift off the bacteria. Surprisingly though, SEM analysis showed intact, uniformly thin long-distance macrophage-bacteria contacts (Fig. 4B) indicating that the filopodium in contact with the bacteria had retracted and thereby had stretched the bacterial fimbriae considerably as compared to the average length of a type 1 fimbrium of about 1 μm^17^. Previous work showed that fimbriae could be extended 8–10 fold due to the uncoiling of their quaternary structure before the receptor-ligand interaction ultimately broke^29^,^30^. Yet, no pickup events of the bacteria from the mannosylated surface were observed at this stage (Fig. 1, 2, Supplementary Movie S1). To enable the uptake, macrophages extended lamellipodia towards the bacteria guided by the firm filopodium–fimbrium hook contacts (Fig. 1, 2). The macrophages finally pushed the lamellipodium underneath the bacteria thereby severing the substrate-adhering fimbriae (Fig. 4C). Once the bacterium is completely separated from the surface, the phagocytic cup is formed and the bacterium internalized (Fig. 1, 2).

## Discussion

**Figure 7:**
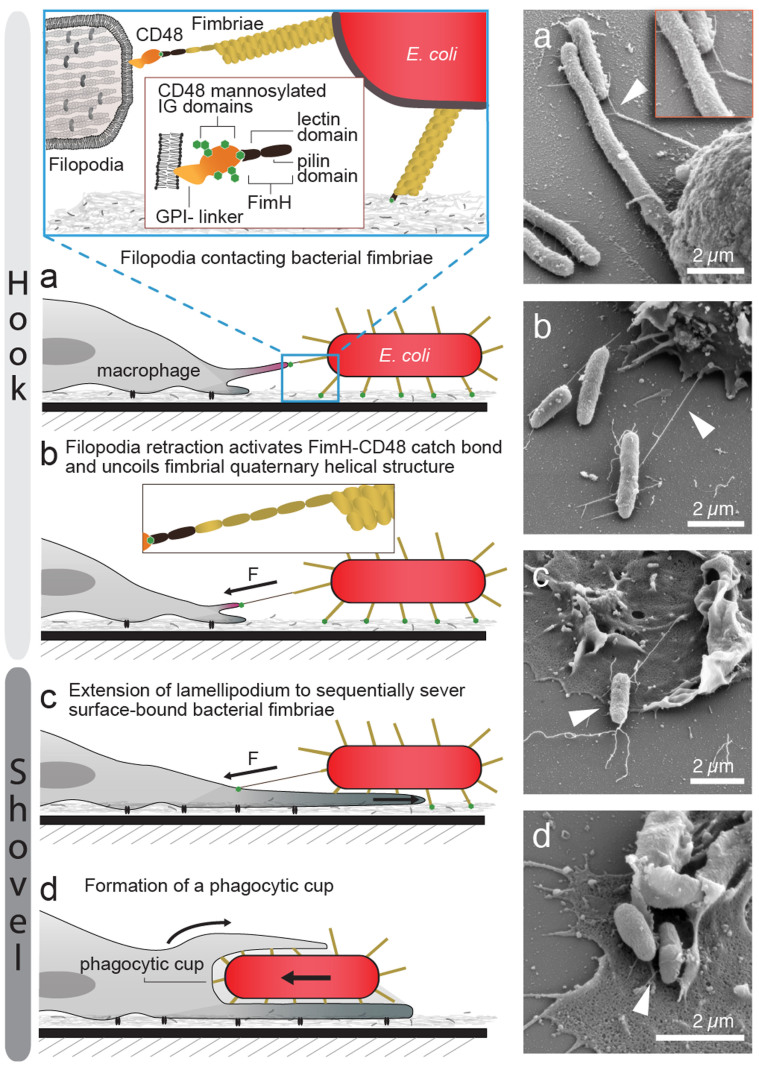
Proposed “Hook-and-Shovel” mechanism for macrophage pick up of surface-bound type 1 fimbriated *E. coli*. (a) The mannosylated membrane anchored surface receptor CD48 of macrophages specifically binds to the bacterial fimbrial tip adhesin FimH, which contains a single mannose-binding pocket in the lectin domain (Hook). (b) Due to filopodia retractions, the bacterial fimbriae are elongated. As bacteria adhere tightly to substrate surfaces via multiple bonds, the macrophages fail to pull bacteria off the surface via the hook alone. (c) To facilitate uptake, macrophages protrude lamellipodia towards the bacterium to sequentially break the bonds that anchor *E. coli* to the surface (Shovel). (d) Once the bacterium is completely lifted off the substrate and lies on the lamellipodium, a phagocytic cup is formed to internalize the bacterium.

Here we analysed the mechanobiological process that enables macrophages to lift-off surface-bound *E. coli*. We highlight how mechanics orchestrate the cooperation of macrophage filopodia and lamellipodia to enable bacterial pick up. Optical imaging combined with electron microscopy allowed us to capture and identify sequential phases of the process (Fig. 7).

In a first phase, macrophages extend and retract filopodia that last from several seconds up to 2 minutes (Fig. 2C). Those transient filopodia enable the macrophages to explore the microenvironment within the filopodia reach (Fig. 1, Fig. 5C, Supplementary Movies 1 and 2). Once a filopodium encounters a bacterium, a specific contact can be formed (hook). In case of type 1 fimbriated *E. coli*, such a hook consists of a single mannose sensitive receptor-ligand interaction between mannosylated CD48 and FimH (Fig. 3, 4). These single filopodium-fimbrium contacts are very stable and can last for up to 40 minutes (max time measured in this study, Fig. 2B). Long-lived interactions are required for the macrophage to protrude a lamellipodium towards the bound bacterium in a second phase of the multistep process that ultimately leads to *E. coli* phagocytosis (Fig. 1, 2).

From the literature it is known that filopodia apply tensile forces to bound objects in the range of pico to nanoNewton^26^,^31^. For many biologically relevant receptor-ligand interactions, e.g. biotin-streptavidin, the bond lifetime is significantly reduced upon exertion of a tensile mechanical force (slip bond)^32^. For single FimH-mannose bonds, we have previously shown that FimH interacting with mannose forms a catch bond^23^,^25^. The bacterial FimH can thereby exist in two structural states that have distinctly different off-rates, both of which have been resolved recently^24^ (Supplementary Table S1). AFM force spectroscopy measurements of single FimH-mono-mannose interactions showed that the dissociation rate of the FimH-mannose catch bond can be reduced by several orders of magnitude after force-activation, i.e. from k_off_ = 1.4 s^−1^ to k_off_ = 5.1*10^−6^ s^−1^ for the short-lived and the force-activated long-lived structural state, respectively^23^,^24^. To verify a catch-bond like adhesion between CD48 and FimH, we performed flow chamber rolling assays of type 1 fimbriated *E. coli* to CD48 coated surfaces (Fig. 6). The CD48-FimH interaction resembles more closely the force-dependent adhesion of FimH to tri- than to mono-mannose^18^ (Fig. 6). To stably bind to surface-bound *E. coli*, macrophages might thus exploit the same catch bond mechanism that allows *E. coli* to adhere to mannosylated surfaces under fluid flow^33^. Our live cell experiments revealed that the existence of those stable contacts kept the filopodia extended several fold longer than in situations where no contacts with a bacterium had formed (Fig. 2B, C). Macrophage filopodia retraction forces might thus activate the mannose-specific single bonds formed between CD48 and FimH to form long-lived macrophage – bacteria interactions. As seen in electron microscope images (Fig. 4C, Fig. 7B), the bacterial fimbriae extend considerably once the filopodia had retracted. Up to ten-fold length increase of type 1 fimbriae due to fimbrial uncoiling was recorded when we previously pulled on catch bond forming fimbriae with a tri-mannose coated AFM tip^29^. The macrophage filopodia thus pull against a soft fimbrial spring rather than a rigid background whose mechanical properties are perfectly tuned to keep the FimH-mannose catch bond activated. In case that the tensile force declines or drops to zero, for example due to force fluctuations, the fimbriae can recoil which slows the back-conversion of the adhesive contact to the short-lived state^23^. Fimbriae uncoiling-and-recoiling might thus contribute to maximize the lifetime of the filopodia-fimbriae contacts^29^.

Stable filopodia contacts consistently stimulated the local protrusion of a lamellipodium which ultimately steered the macrophage to move towards the bacterium (Fig. 1, 2). The lamellipodium protrusion rate is regulated by the actin polymerization rate at the leading edge^34^. This local protrusion force is counterbalanced by the uniform tension of the cell membrane^35^. In J774A.1 macrophages, as used in this study, applying local centripedial forces exceeding 0.5 nN can induce conical protrusions that resemble lamellipodia^36^. Furthermore, the mode of filopodia adhesion was shown to modulate lamellipodia protrusion in fibroblasts and neurons^27^,^37^,^38^. While the detailed mechanisms by which tensile forces stimulate lamellipodium protrusions are not yet known, our findings support various observations in the literature indicating that tensile mechanical forces play a central role in regulating events associated with actin polymerization, i.e cell migration and spreading^39^,^40^,^41^.

Since bacteria adhere to surfaces with multiple bonds^28^, rupturing them all at once requires considerably higher forces than if such a cluster of bonds is broken sequentially^32^. Since only moderate forces in the pico to nanoNewton range can be generated by macrophages retracting a filopodium, breaking the multiple bacterium-surface interactions simultaneously by just pulling on the filopodium-fimbrium hook is very unlikely to occur (summary see Supplementary Table S1). Here, we observed that *E. coli* pick up from the surface typically occurs when a macrophage pushes its lamellipodium underneath the bacterium (Fig. 1, 4). The lamellipodium could thus act as a shovel, suggesting that the multiple bacterium-surface bonds are broken sequentially, i.e. in a zipping mode bond-by-bond (Fig. 1, 4C). We thereby presume that the FimH-mannose contacts are broken since previous work indicated that the mannose-FimH bonds breaks at forces that are too small to fracture the type 1 fimbriae or to pull them out of the bacterial membrane^29^. Alternatively, the adhesive bacterium-substrate interactions might also be broken in sequence if the macrophage manages to peel off the bacterium by pulling with the lamellipodium at an angle, however, we could not record such cases. After the bacterium is lifted-off the surface, a phagocytic cup is formed and bacterial uptake is accomplished (Fig. 1, 2). Interestingly, a similar lamellipodia based shovel-like mechanism, exerting picoNewton forces, was reported for the removal of surface-bound obstacles by neuronal cells^42^,^43^.

In conclusion, our data identifies the sequential mechanobiological process that macrophages exploit to clear surface-bound *E. coli*. We highlight that the kinetics and mechanical properties of the macrophage filopodia, lamellipodia as well as the *E. coli* fimbriae have to be tightly tuned to each other to facilitate bacterial uptake. Future research might explore whether the process described here is unique to the CD48-FimH system, or whether other receptor-ligand interactions between immune cells and bacteria exist that could potentially be exploited in similar ways for the uptake of other pathogens. With respect to the CD48-FimH mediated uptake, it was described that fimbriated *E. coli* that had been phagocytosed by macrophages could partially survive within the cells for at least one hour^44^. It was therefore suggested that the FimH entry mechanism might be associated with colonization of macrophages, rather than being an effective host defence. More recently though, it was shown that microbes that enter macrophages through a cholesterol-sensitive pathway, like FimH expressing *E. coli* binding to CD48, avoid immediate digestion in lysosomes by interacting with the autophagy pathway of the host cells^45^. In contrast to the previous interpretation that bacteria might use the CD48 pathway to actively invade macrophages^44^, it is now suggested that prolonging the survival of bacteria in autophagosomes could promote the presentation of native antigens in naïve hosts prior to their final disposal in the lysozymes^45^. Rather than rapid killing and digestion of pathogens, extending the lifetime of bacteria inside the macrophage might boost the adaptive immune response. It might allow macrophages to increase the expression of major histocompatibility molecules (MHC) crucial for antigen presentation upon inflammation^46^,^47^. Furthermore, recent *in vitro* and *in vivo* studies have shown that FimH induces a potent innate antiviral response in murine macrophages and in primary mouse embryonic fibroblasts, which is predominantly mediated by the production of type I interferon^48^. Finally, our study suggests for the first time that soluble inhibitors that are currently exploited to suppress bacterial infections, might instead have an unanticipated adverse effect by protecting firmly adhering *E. coli* from being sensed and cleared by the natural host immune cells (Fig. 5).

## Methods

### Bacteria

Recombinant *E. coli* FimH-j96 bacteria were constructed by transforming the *fim* null K-12 derivative AAEC191A^49^ with the plasmid pSH2 (kind gift from Prof. P. Orndorff, NCSU). *E. coli* FimH-f18 were constructed as described previously^24^. Bacteria were grown for 16 h in LB medium at 37°C/150 rpm supplemented with 25 μg/ml chloramphenicol and adjusted to OD_670 nm_ = 0.3 in PBS (~10^9^ cfu/ml, Biorad SmartSpec™ Plus).

### Cell culture

J-774A.1 macrophages (DSMZ ACC-170) were cultured as exponentially growing subconfluent monolayers at 37°C/5% CO_2_ in DMEM (Gibco 21885) supplemented with 10% (v/v) heat inactivated FBS (Sigma Aldrich F7524) and 1% (v/v) penicillin/streptomycin (Sigma Aldrich P0781). Cells were detached with a rubber policeman prior reaching confluence, centrifuged at 200 g and resupended in fresh 37°C DMEM + 10% FBS.

### Phagocytosis assay

Live-cell correlation experiments were performed in 4-well Labtek dishes (Thermo Scientific 155382) while all other experiments were done in flow chambers (0.1 ml/min flow) to ensure constant media exchange. Surfaces were coated with a 1:1 mixture (v/v) of 100 μg/ml RNaseB (Sigma Aldrich R1153) and 100 μg/ml purified human plasma FN (purification procedure as described previously^50^) for 1 h. Bacteria were allowed to adhere for 10 min in 37°C DMEM + 10% FBS prior to macrophage injection (25.000 cells/ml). For inhibitor studies, the culture media was supplemented with 2% (w/v) methyl α-D-mannopyranoside (αMM, Sigma Aldrich M6882) or the macrophages were preincubated for 1 h with 1:4 diluted CD48 antibody (abcam ab48313). Live cell DIC microscopy was performed for up to 40 minutes using a Nikon TE2000-E microscope equipped with a 37°C incubation chamber. Images were acquired with a Hamamatsu EM CCD-9100 camera using Metamorph™ (Molecular Devices, Inc.).

### Bacterial accumulation assays

35 mm tissue culture dishes (Corning CellBIND, 3294) were incubated for 75 min at 37°C with either 100 μg/ml mono-mannose-BSA (Dextra Labs, NGP1108), 20 μg/ml RNaseB (Sigma Aldrich, R1153) or 20 μg/ml recombinant mouse CD48 (R&D Systems, 3327-CD) in 0.02 M bicarbonate buffer. After blocking with 0.2% PBS-BSA for 30 min, the dishes were incorporated into a parallel plate flow chamber (Glycotech). Bacteria were resuspended in 0.2% PBS-BSA to a final OD_670 nm_ = 0.03 (~10^8^ cfu/ml). Media flow was adjusted with a syringe pump (Harvard Apparatus). The number of accumulated bacteria at the given shear stresses was analysed from 5 min time-lapse videos as described previously^24^. The exact 95% confidence intervals for a Poisson variable were used as error bars. The fraction of firm adhering bacteria was determined from the last 1 min of the accumulation videos. By subtracting the intensity values in each pixel in the second image from the first, rolling bacteria (moved at least one-half bacterial diameter over 30 s) appeared as a pair of dark and light spots, which were counted using a threshold and an automated cell counting package. The remaining bound bacteria were assumed to be stationary. For the fraction of firmly adhering bacteria, the exact 95% confidence intervals for a binomial variable were used.

### Fluorescence confocal microscopy

After fixation in 4% (v/v) PFA-PBS for 20 min, samples were permeabilized with 0.1% (v/v) Triton X-100 (Sigma Aldrich T8787) for 10 min prior to blocking with 5% (v/v) donkey serum (DS, Millipore S30)/2% (w/v) BSA (Fluka 05470) - PBS for 1 hour. 1:50 diluted Goat anti *E. coli* antibody (Serotec, OBT0986) and 1:200 diluted Donkey anti Goat Dylight 649 secondary antibody (Jackson, 705-495-147) in 5% DS/2% BSA-PBS were used according to suppliers instruction. For actin staining, 1:100 diluted AlexaFluor 488 phalloidin (Invitrogen, A12379) was added for the last 15 min of the secondary antibody incubation. Samples were postfixed in 4% PFA for 10 min and mounted in Prolong Antifade Gold (Invitrogen, P36930) for 48 hours prior to data acquisition. Samples were analysed using a high-resolution Olympus FV1000 confocal microscope using a TIRFM 60× 1.45 NA oil immersion objective. Deconvolution was performed with Huygens software (SVI, Hilversum, Netherlands) using fluorescent microspheres (Invitrogen, P7220) as calibration. Image processing was performed using IMARIS software (Biplane AG, Zurich, Switzerland).

### CD48 staining

Samples were washed with 4°C PBS, blocked for 15 min with 4°C 5% DS/2% BSA-PBS and incubated with 1:10 diluted monoclonal biotinylated CD48 antibody (abcam ab95606) in 5% DS/2% BSA-PBS for 30 min on ice. After washing 3 times 5 min with 4°C PBS, 1:100 diluted ExtrAvidin-Cy3 conjugate (Sigma Aldrich E4142) in 4°C 5% DS/2% BSA-PBS was applied prior to sample fixation in 4% PFA-PBS for 15 min at room temperature.

### SEM sample preparation

Samples were consecutively fixed in 4% (v/v) formaldehyde and 2% glutaraldehyde - PBS for 30 min, followed by 2% OsO_4_ incubation for 20 min. Samples were dehydrated in a graded series of ethanol and dried over the critical point of CO_2_ (T_k_ = 31°C, P_k_ = 73, 8 bar, CPD 030, Bal-Tec AG, Balzers, Liechtenstein). After sputter coating with 10 nm platinum images were recorded with a Zeiss SUPRA 50 VP using secondary electron signals.

### Bacterial displacement analysis

Displacements of the surface-adhering *E coli* upon macrophage contact were calculated from the x, y coordinates of the bacterial pole (dashed line Fig. 2) based on 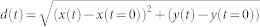.

## Author Contributions

J.M., T.L., M.C., H.H. and V.V. designed experiments. J.M. and T.L. performed experiments and analysed data. J.M., T.L., M.C. and V.V. wrote the manuscript.

## Supplementary Material

Supplementary Information Supplementary Information

Supplementary Information Supplementary Movie 1

Supplementary Information Supplementary Movie 2

Supplementary Information Supplementary Movie 3

Supplementary Information Supplementary Movie 4

Supplementary Information Supplementary Movie 5

Supplementary Information Supplementary Movie 6
