# Supplementary material for: Macrophages lift off surface-bound bacteria using a filopodium-lamellipodium hook-and-shovel mechanism
Source: Sci Rep. 2013 Oct 7;3:2884. doi: 10.1038/srep02884 (PMC3791455; doi:10.1038/srep02884)
Supplement: Supplementary Information [file srep02884-s1.pdf]

Macrophages lift off surface-bound bacteria using a hook-and-shovel mechanism

Jens Möller, Tessa Lühmann, Mamta Chabria, Heike Hall, Viola Vogel

Supplementary Information

Supplementary Table S1

experiment	key findings	ref
**Macrophage (J774A.1 and RAW 264.7) filopodia retraction force-velocity relationship measured by optical tweezers**	- Stepwise retraction of filopodia with a mean step size of 36 nm implied molecular motor activity<br>- Pulling forces of up to 19 pN were recorded<br>- Pulling velocities were strongly dependent on counter-acting forces and ranged between 600 nm/s at forces < 1 pN and approximately 40 nm/s for forces > 15 pN	(26)
**Micromechanics of J774A.1 macrophage filopodia measured by magnetic bead displacement tracking**	- filopodia can create large retraction forces of up to 0.5 nN over distances > 10 µm<br>- filopodia retraction velocities up to 160 µm/s were observed	(31)
**Impact of force on lifetime of non-covalent receptor-ligand interactions**	- lifetimes of non-covalent single slip bonds can be drastically reduced by externally applied force	(32)
**Biophysics of catch bonds**	- mechanical force can cause a structural switch from a short-lived to a long-lived receptor-ligand interaction. Kinetic and structural data revealed that the FimH-mannose catch bond complex consists of two distinct structures and is allosterically activated by force<br>- review of a few common catch bond interactions found among adhesion molecules	(14)
**Single molecule const. velocity AFM studies on FimH-mannose catch bond activation**	- FimH-mannose dissociation rate is reduced by six orders of magnitude after force-activation, i.e. from k_{off} = 1.4 s^{-1} to k_{off} = 5*10^{-6} s^{-1} for the short-lived and the force-activated states, respectively	(23)
**Single molecule const. force AFM studies on FimH-mannose catch bond activation**	- the short-lived bonds dissociate within 1 s for constant tensile forces < 20 pN<br>- at a constant tensile force < 70 pN, up to 70 % of the FimH-mannose interactions became long-lived	(24)

**Uncoiling mechanics of *E. coli* type 1 fimbriae are optimized to enhance survival of FimH-mannose catch bonds**	- mechanics of the type 1 fimbrial rod regulates the lifetime of the FimH-mannose catch bond<br>- individual fimbriae elongate several fold, up to 10 μm at forces > 60 pN, by uncoiling of the quaternary structure of the helical fimbrial shaft<br>- fimbriae coil back at forces < 25 pN<br>- fimbriae act as dampers of force fluctuations	(29)
**Catch bond behavior is impacted by the mannose motif presented to the FimH**	- FimH adhesion to oligomannose motifs in contrast to monomannose ligands forms catch bonds as well, as additional mannose rings bind to the outside and around the binding pocket of the lectin domain, but lacks the characteristic shear threshold for binding at low forces	(18)

Supplementary Table S1. Summary of nanomechanical measurements on macrophage filopodia, bacterial type 1 fimbriae mechanics and of the lifetimes of single FimH-mannose catch bonds.

Supplementary Movie captions

Supplementary Movie 1. High resolution DIC live cell time-lapse movie of macrophage filopodia (FP) exploring the FN-RNaseB coated substrate. If a filopodium encountered an *E. coli*, a stable contact was formed that remained intact and allowed the macrophage to locally protrude a lamellipodium (LP) towards the surface bound bacterium. Once the LP had reached the bacterium, membrane locally accumulated in front before being pushed underneath the bacterium.

Supplementary Movie 2. DIC live cell time-lapse movie of macrophages first screening the surface and then exploiting a multiphase “Hook-and-Shovel” mechanism to pick up fimbriated *E. coli* from the substrate. Independent of fluid flow direction, filopodia protrude from the macrophage membrane and screen the local environment to sense surface-bound type 1 fimbriated *E. coli*. Stable filopodia–bacteria contacts are observed. Mediated by the filopodia-bacteria contacts, macrophages locally protrude lamellipodia towards the bacteria.

Uptake events of surface-bound bacteria were observed when the lamellipodia could reach underneath the entire bacteria. This was followed by a rapid remodeling of the plasma membrane to form the phagocytic cup to facilitate uptake.

Supplementary Movie 3. DIC live cell time-lapse movie of a macrophage exploiting a multiphase "Hook-and-Shovel" mechanism to pick up fimbriated *E. coli* from surfaces. Long-lived macrophage filopodium-bacterium interactions exceeding 40 minutes are observed. For successful phagocytic uptake, the macrophage locally protruded lamellipodia to lift the bacteria off the surface. By actin-dependent remodeling of the macrophage cell membrane, a phagocytic cup was formed and the membrane is protruded over the bacterial targets.

Supplementary Movie 4. DIC live cell time-lapse movie of a macrophage filopodium and lamellipodium encountering a surface-bound *E. coli*. Filopodium-bacterium contact remained intact for 14 minutes. Bacterium displacement from its original position was only observed after it was contacted by a lamellipodium. Once the lamellipodium completely engulfed the bacterium, the uptake occurred with a speed of 1.6 μm/min.

Supplementary Movie 5. DIC live cell time-lapse movie of a macrophage lamellipodium directly encountering surface-bound *E. coli*. After complete lamellipodium engulfment, bacterial uptake proceeded with 1.7 μm/min min indicating that the underlying phagocytic mechanism is common for a lamellipodium-mediated uptake with our without previous filopodia contact (see Supplementary Movie 4).

Supplementary Movie 6. DIC live cell time-lapse movie of a macrophage filopodium encountering surface-bound *E. coli*. The filopodium-bacterium contact remained stable

for 7 min before it broke. The filopodium contact alone was not sufficient to displace the surface-bound *E. coli*.
